# Outcome unpredictability affects outcome-specific motivation to learn

**DOI:** 10.3758/s13423-021-01932-x

**Published:** 2021-05-04

**Authors:** Genisius Hartanto, Evan Livesey, Oren Griffiths, Harald Lachnit, Anna Thorwart

**Affiliations:** 1 Department of Psychology, Philipps-University Marburg, Gutenbergstrasse 18, 35052 Marburg, Germany; 2 University of Sydney, Camperdown, Australia; 3 Flinders University, Bedford Park, Australia

**Keywords:** Outcome predictability, Outcome-specific motivation, Active learning, Predictive learning, Learned helplessness

## Abstract

Outcome predictability effects in associative learning paradigms describe better learning about outcomes with a history of greater predictability in a similar but unrelated task compared with outcomes with a history of unpredictability. Inspired by the similarities between this phenomenon and the effect of uncontrollability in learned helplessness paradigms, here, we investigate whether learning about unpredictability decreases outcome-specific motivation to learn. We used a modified version of the allergy task, in which participants first observe the foods eaten by a fictitious patient, followed by allergic reactions that he subsequently suffers, some of which are perfectly predictable and others unpredictable. We then implemented an active learning method in a second task in which participants could only learn about either the previously predictable or unpredictable outcomes on each trial. At the beginning of each trial, participants had to decide whether they wanted to learn about one outcome category or the other. Participants at the beginning of the second task chose to learn about the previously predictable outcomes first and to learn about the previously unpredictable outcomes in later trials. This showed that unpredictability affects future motivation to learn in other circumstances. Interestingly, we did not find any sign of outcome predictability effect at the end of the second phase, suggesting that participants compensate for biased outcome sampling when making overt choices in ways that they may not when learning about both outcome categories simultaneously.

The outcome predictability (OP) effect refers to the tendency to learn better about an outcome that has a history of predictability than about an outcome with a history of unpredictability, even if both are encountered in new situations where all outcomes are predictable. For instance, take a commonly used causal learning task (the allergy task) in which the learner is presented with a fictitious patient, Mr. Y, suffering from food allergies. When Mr. Y eats shrimp, he always suffers from skin itchiness. The learner can then use eating shrimp as a cue to predict skin itchiness, a thereby predictable outcome. In contrast, Mr. Y eating peanuts sometimes results in stomach bloating and sometimes stomach cramps. This makes both stomach bloating and stomach cramps unpredictable outcomes. In a new situation, even if these allergic reactions are fully predictable, the learner would learn better to predict skin itchiness than stomach bloating or cramps.^1^

Four different predictive learning protocols have investigated the OP effect (Griffiths et al., 2015; Griffiths et al., 2018; Liu et al., 2020; Quigley et al., 2017; Thorwart et al., 2017), and a meta-analysis reported that the OP effect is significantly evident across these protocols (Griffiths et al., 2019). The OP effect was reported only recently and has been documented in relatively few studies to date. Naturally, questions about the characteristics and underlying mechanism of the OP effect remain. The current experiment investigated whether the OP effect is due to changes to outcome-specific motivation to learn by outcomes’ prior predictability.

This hypothesis is based on the similarities between the OP effect and the learned helplessness (LH) effect (Maier & Seligman, 1976, 2016). The LH effect describes the tendency to learn better about an outcome that has a history of controllability than an outcome that has a history of uncontrollability, even if both outcomes are encountered in a new situation where all outcomes are controllable. The outcome in LH tasks refers to a stimulus that can or cannot be altered by a response of the learner. For example, an LH procedure in which a tone can be stopped by pressing a button makes the tone controllable, while a tone that cannot be stopped is uncontrollable. Several features of LH and the OP effect are similar. First, learning in an initial phase establishes a bias that is observed in subsequent learning and behavior. Although they differ in terms of generalizability—LH appears to transfer to very different tasks that involve new learning materials, whereas the OP effect involves learning about the same outcome in two different situations—in both cases, initial learning results in a relative deficit in later learning and performance. Second, outcome predictability is a major component of both manipulations. Using the previous example, when participants can successfully stop a tone, it is also naturally predictable as it can be predictably stopped. In contrast, uncontrollable tones are also unpredictable as the stopping of the tones in each trial is controlled by some hidden process. Some studies have investigated the role of both predictability and uncontrollability in the LH effect. Burger and Arkin (1980) investigated the roles of perceived predictability and perceived controllability by separating the two properties of the outcomes to some degree. Only participants who were exposed to both uncontrollable and unpredictable tones showed a sign of LH effect in a new situation. They, therefore, concluded that not only the perceived controllability but also perceived predictability is crucially involved in determining LH effects, thereby linking the LH effect directly to the outcome unpredictability in OP protocols. Third, both effects have the element of failure in the first of their tasks. In the anagram LH task by Hiroto and Seligman (1975), for example, participants “fail” to solve the anagrams in the first situation. Similarly, participants “fail” to make correct predictions of an outcome in the first situation of OP effect protocols.

Given the similarities, we argue that both the OP effect and the LH effect share an underlying principle: demotivation to learn as a result of exposure to unpredictability. According to accounts of the LH effect, the learning bias is mainly based on the changes to the motivation to learn (Burger & Cooper, 1979; Miller & Seligman, 1976). Expectancy-value theory (EVT) (Eccles & Wigfield, 2002) explains that motivation is based on the individual’s expectancies of how probable a result can be achieved and the value that the individual places on the desired result. These expectancies are influenced by task-specific beliefs, such as perceptions of the controllability of the task. In LH tasks, the perception of uncontrollability over one outcome like an aversive tone will form an expectancy that the responses will not create the desired result of stopping the tone (Miller & Seligman, 1973), and this may decrease one’s motivation for further engagement in future tasks involving similar outcomes and situations (Alloy & Abramson, 1982).

However, despite these links between controllability and predictability, there is relatively little known about the relationship between the unpredictability of a specific outcome and motivation to engage and learn about that particular outcome in OP tasks. In OP tasks, the perception of unpredictability will form an expectancy that predictive responses will not create the desired result of correct predictions, and this may decrease one’s motivation for further engagement in future situations involving that particular outcome.

Based on this argument, we investigated the relationship between outcome-specific motivation and OP using an active learning method. Active learning refers to learning procedures in which learners can actively seek information (Kruschke, 2008). Kruschke (2008) described an example procedure that lets the participants choose at the beginning of each trial which cues would be more informative to learn about. Their choices, therefore, represent their motivation to learn about that specific cue. In our task, we were interested in measuring the motivation to learn about a specific outcome category instead. Therefore, we adapted the active learning procedure by having participants choose which specific outcome category they wanted to learn in each trial of the second learning situation. If the unpredictability of an outcome in the first situation leads to a motivational deficit, we expect that participants are more eager to learn first about the previously predictable outcomes in the second situation.

## Methods

### Design

The design of the experiment is summarized in Table 1. Our behavioral experiment used a modified version of the allergy task described by Griffiths et al. (2015). In this two-phase learning task, participants were shown either one or two vegetables or fruits (cues) and were told about the allergic reactions Mr. X suffered (outcomes) when he ate those foods. The first phase familiarized the participants with the two outcome categories: predictable (p) and unpredictable (u), represented as either skin-related or stomach-related allergic reactions. As shown in Table 1, Outcome p1 would always occur when cue Vegetable A was presented. Similarly, p2 would always occur when cue Vegetable B was presented. A third vegetable (X) preceded unpredictable outcomes. For half of the trials, Outcome u1 would occur when cue X was presented, and the other half u2. In the second phase, a new set of cues (fruits) was introduced: E, F, G, and H, but the outcomes remained the same as Phase 1. Each cue in Phase 2 was predictive of one outcome in each category. Crucially, at the beginning of each trial in this second phase, and before participants saw which fruits Mr. X had eaten in this trial, participants had to choose which outcome category they would like to learn. If they chose, for example, skin reactions, they would only receive information about the skin-related reaction Mr. X suffered after eating one of the fruits. The same rule was applied for stomach-related reactions.

**Table 1:** Experimental design

Phase 1	Phase 2	Test
A → p1, u0	EY → p1 or u2	E?
B → p2, u0	FY → p2 or u1	F?
X → u1, p0	GY → p1 or u1	G?
X → u2, p0	HY → p2 or u2	H?
AX → p1, u1		Y?
AX → p1, u2		
BX → p2, u1		
BX → p2, u2		

*Note.* Letters A–Y represent foods (A, B, and X: vegetables; E, F, G H, and Y: fruits), and the symbols [p0, p1, p2, u0, u1, u2] represent allergic reactions. The letters “p” and “u” represent the predictable category and the unpredictable category, respectively. For instance, if skin-related reactions are the predictable category, then p0 would refer to “no skin reaction,” p1 to “skin swelling,” and p2 to “skin itchiness.” For the unpredictable outcome category, u0 would refer to “no stomach reaction,” u1 to “stomach bloating,” and u2 to “stomach cramps.”

### Participants

Twenty undergraduate students of the Philipps-Universität Marburg completed the experiment. The sample consisted of 17 identifying as female, two identifying as male, and one did not specify their gender. Age data were collected for 11 participants (the age of the remaining participants was lost due to technical problems). The age of the collected participants ranged from 18 to 30 years (*M* = 23.27, *SD =* 3.66). They were paid with money or received course credit.

Using G*Power (Faul et al., 2007), a sensitivity analysis (two-sided, α = .05, power = 80%) for a one-way analysis of variance (ANOVA) of the choices in Phase 2 revealed a minimal detectable effect (MDE) of *f* = .19, an effect of medium size. (As we report contrasts, the actual power was higher or the actual MDE was lower; see Lazic, 2018). A sensitivity analysis for the follow-up *t* tests resulted in an MDE of *d* =.66. Therefore, given the sample size of 20 participants, we were able to detect medium to large effects.

### Apparatus and stimuli

This experiment was developed using JavaScript and HTML language, and the JsPsych plugin (https://www.jspsych.org/; de Leeuw, 2015). Five vegetables served as cues during the first phase: broccoli, carrots, mushrooms, corn, and green peas. The assignments of these vegetables to the cues A, B, or X were randomized for each participant. In the second phase, five fruits were randomly assigned to the five cues E, F, G, H, and Y: apple, apricots, banana, grapes, and strawberries. Two outcome categories with three choices each were presented on the screen as six rectangles in the middle of the screen, with different colors for each button. The six possible outcome buttons were labeled as: “no skin reaction,” “skin swelling,” “skin itchiness,” “no stomach reaction,” “stomach bloating,” and “stomach cramps.” The allocation of the outcome categories was counterbalanced across participants. Half of the participants experienced skin reactions as predictable and stomach reactions as unpredictable. The other half experienced skin reactions as unpredictable and stomach reactions as predictable.

### Procedure

In the instructions, the participants were told to assume the role of an allergist examining the allergic reactions^2^ of an imaginary patient, Mr. X, after he had eaten vegetables (Phase 1) or fruits (Phase 2).

#### Phase 1

The first phase consisted of 16 blocks of each trial mentioned in Table 1. These trials were randomized within each block. The positions of the cues in each trial on the screen were counterbalanced. When the participants were told that Mr. X had eaten the corresponding vegetable(s), they were asked which allergic reactions Mr. X would have. They did it by clicking two buttons, one button from each outcome category. For example, when Mr. X ate carrots, they may have guessed that he had experienced no skin reaction and stomach bloating by pressing the “no skin reaction” button and the “stomach bloating” button. As soon as they clicked both buttons, they received feedback on whether their predictions were correct or incorrect for each outcome category separately and which two allergic reactions Mr. X suffered.

#### Phase 2

Participants were instructed that they would now learn about the allergic reaction Mr. X suffers when eating fruits, and that they would have to choose which symptom category they would like to learn about. Phase 2 consisted of 12 blocks of each trial type shown in Table 1. The positions of the cues were counterbalanced, and trials were randomized within each block. Before each trial, a question appeared asking the participant, “Which symptoms/reactions do you want to learn more about?” Then the participants had to choose one out of two options: skin-related or stomach-related reactions. Only the three buttons of the chosen category were then presented on the screen together with the cues, and participants were asked to make a prediction by clicking on one of them. They then received feedback about this prediction and the correct allergic reactions of the chosen outcome category.

#### Test phase

After Phase 2, participants were instructed to rate the likelihood of each cue presented in Phase 2 (E, F, G, H, and Y) to cause each allergic reaction (p0, p1, p2, u0, u1, and u2).

#### Manipulation check

At the end of the experiment, the participants were asked to give a rating between 0 and 100 on how confident they were to predict the allergic reactions of Mr. X after eating vegetables (i.e., in Phase 1). This was performed to see if the experimental conditions established in the first phase had worked as expected.

### Data analysis

In the result figures, data normalization based on Cousineau (2005) was performed for the standard error of means (*SEM*s) to better reflect the within-subject design. Significance levels after Greenhouse–Geisser correction of the degree of freedom were reported.

## Results

### Phase 1

**Fig. 1:**
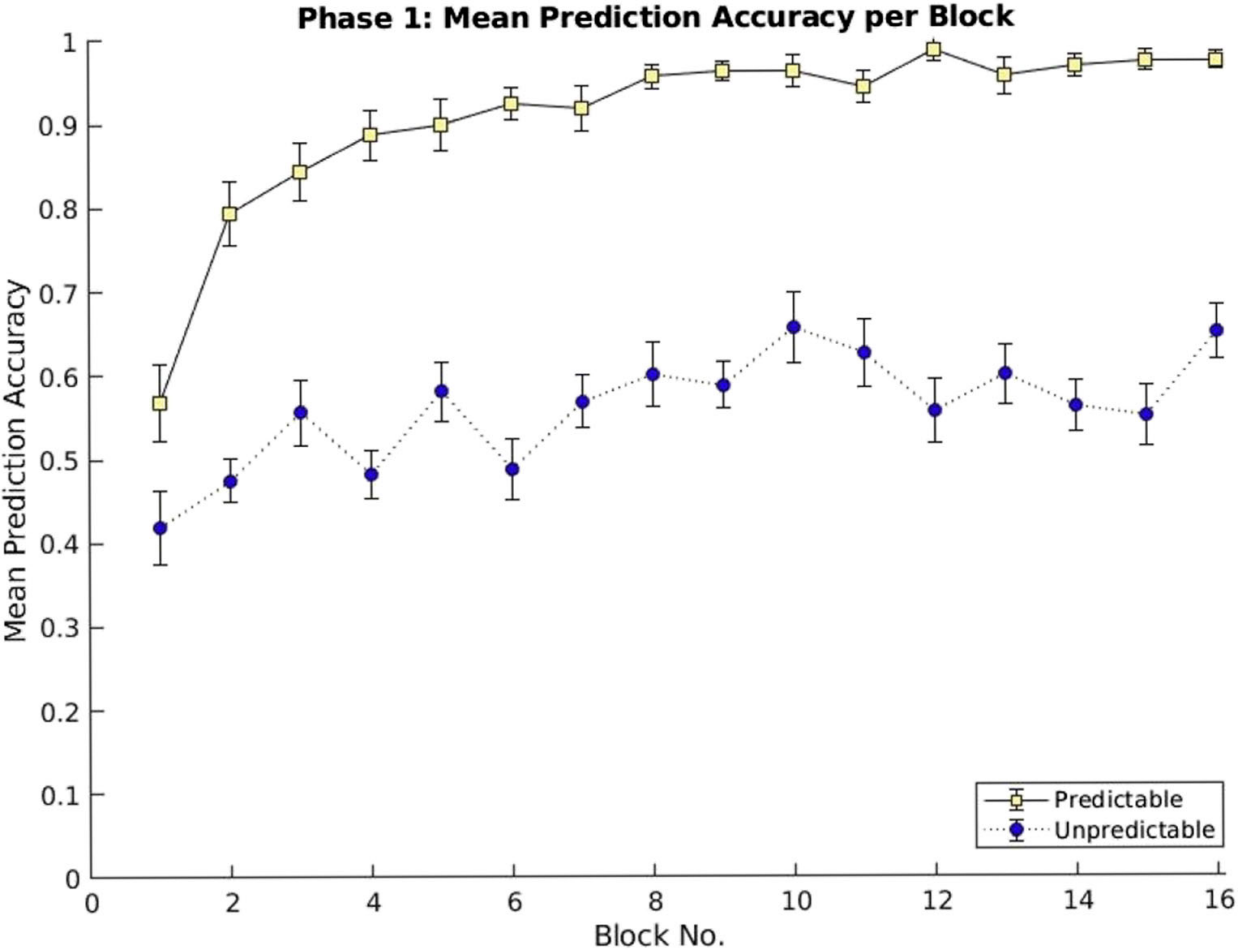
Mean prediction accuracy of Phase 1 averaged across all trials of each block. The black line with light-yellow data points represents the predictable outcome category, and the dotted line with blue data points represents the unpredictable outcome category. Error bars indicate the *SEM* of the normalized data. (Color figure online)

The mean prediction accuracy, calculated as the proportion of correct predictions, increased according to the outcomes’ predictability (see Fig. 1). A repeated-measures ANOVA, with two within-subject factors, Predictability (Predictable and Unpredictable) and Blocks (1–16), confirmed significant main effects, Predictability: *F*(1, 19) = 246.390, p < .001, η_p_^2^ = .928; Blocks: *F*(1, 19) = 13.217, *p* < .001, η_p_^2^ = .410, and a significant interaction: *F*(15, 285) = 3.268, *p* = .002, η_p_^2^ = .147.

### Phase 2

**Fig. 2:**
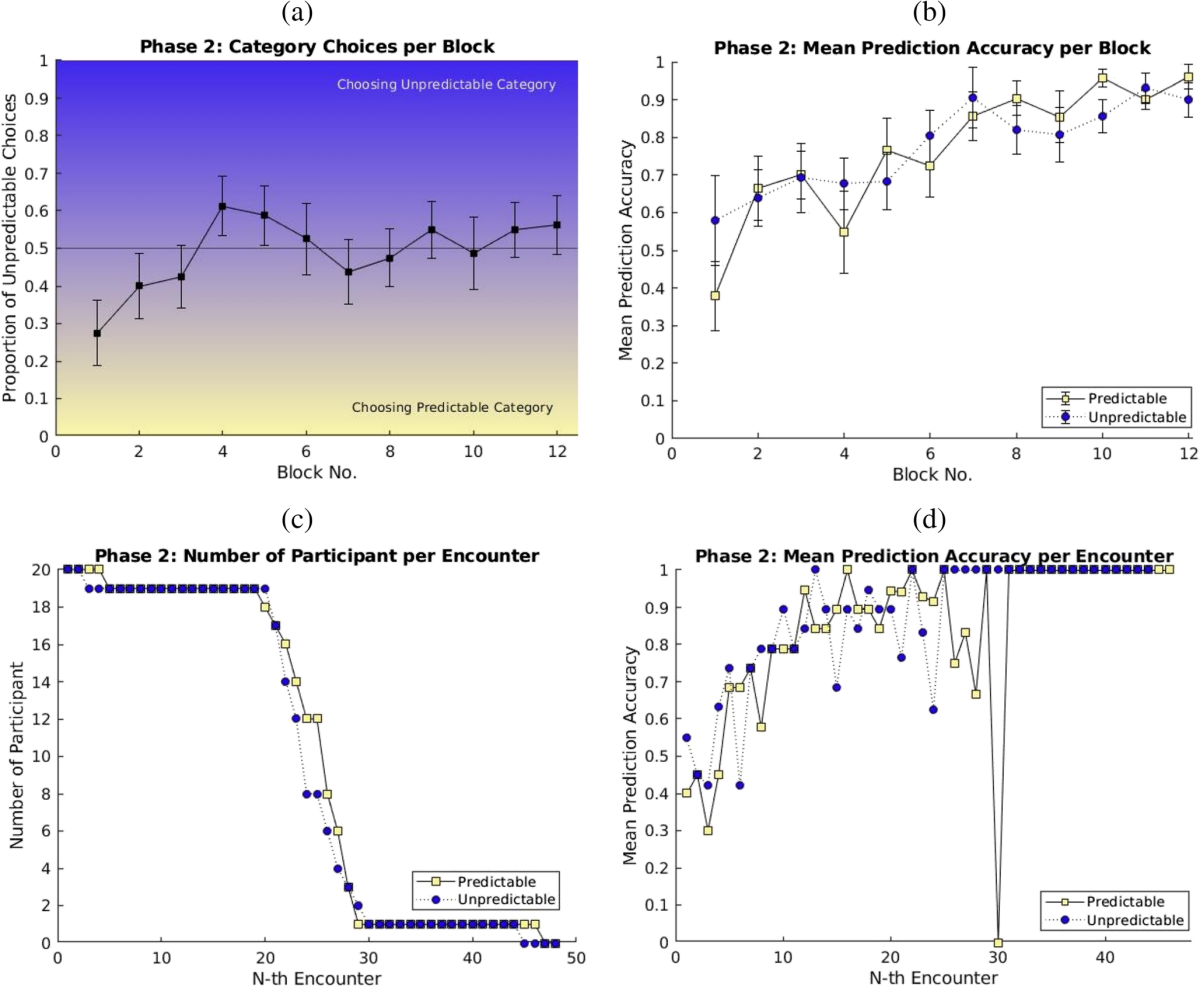
**a** The proportion of unpredictable choice for each block. The line at 0.5 indicates the mid-point between the choices. The blue shade represents the choice of unpredictable category, and the yellow shade represents the choice of predictable category. **b** Mean prediction accuracy of Phase 2 averaged across all trials of each block. **c** The number of participants at the *Nth* outcome encounter (outcome category that had been experienced). **d** Mean prediction accuracy of Phase 2 averaged across the *Nth* outcome encounter. In Panels **b–d**, the black lines with light-yellow data points represent the previously predictable outcome category, and the dotted lines with blue data points represent the previously unpredictable outcome category. Error bars indicate the *SEM* of the normalized data. (Color figure online)

Figure 2a shows the mean proportion of choices of the previously unpredictable outcome across the four trials in each block. Values below 0.5 indicate that the previously predictable outcome category was chosen more often. Using a one-way ANOVA, with repeated-measure factor Blocks (1–12) and polynomial contrasts, we found a significant linear trend across blocks, *F*(1, 19) = 5.618, *p* = .029, η_p_^2^ = .228. Follow-up one-sample *t* tests compared the proportion of unpredictable choices to 0.5 for each block. The first block was found to be significantly lower than 0.5, *t*(19) = −3.111, *p* = .006; the remaining blocks did not differ significantly from .5 (*t*s < 1.308, *p*s > .206). This result demonstrates that the participants’ choice was biased towards the previously predictable outcome category at the beginning of Phase 2 (Block 1), with a linear trend shifting towards the other category later. By the end of Phase 2 (Block 12), the participants chose to learn about both categories equally often.

We also checked the average number of trials that were experienced per cue per outcome category (previously predictable versus previously unpredictable) across the entire Phase 2. Because participants could choose which outcome category to learn about on each trial, we could not force equivalence of exposure between outcome categories. A repeated-measures ANOVA, with factors Cue and Outcome Category, found neither a main effect of Outcome Category, *F*(1, 19) < 1, *p* = .786, η_p_^2^ = .004, nor a significant interaction between Outcome Category and Cues, *F*(3, 57) < 1, *p* = .523, η_p_^2^ = .037. There was no evidence that participants saw more trials of one cue–outcome category combination than of the others during Phase 2.

Figure 2b shows the corresponding mean prediction accuracy per block during Phase 2. No differences in accuracy were observed between the previously predictable and unpredictable outcome categories. However, the mean prediction accuracy is not as representative for learning about each outcome as it may seem, as it neglects the participants’ choices for each outcome category. The block-by-block data shown in Fig. 2b can only show the prediction accuracy of trials in which participants chose that outcome category. As the number of participants who chose each outcome category differed, each data point is based on a different number of predictions. Secondly, two participants might choose the previously predictable outcome category in Block 3 but differ concerning what they chose in Blocks 1 and 2. They would therefore differ concerning the opportunity to learn before making their predictions in this block. Moreover, these differences were systematic, rather than random; people had more opportunities to learn about predictable outcomes than the unpredictable outcomes in the first block as their choice was biased at the beginning of Phase 2.

To address these limitations, we reanalyzed the predictions. We compared the accuracy for predictable and unpredictable outcomes on each trial based on the number of previous encounters with each outcome category that had been experienced before that trial, rather than on the block in which that trial chronologically occurred. To make sure that there were enough encounters with each category to meaningfully analyze the data in this manner, Fig. 2c plots the number of participants who had *N* fewer encounters with each outcome category. The inflection point is approximately 25 encounters, suggesting that across Phase 2 most participants had between 24 and 27 encounters with each outcome category. Figure 2d confirmed the previous impression that prediction accuracy in both categories over these encounters increased equally. As the analyses neglect both the varying number of participants, how many previous trials they received, or when the predictions were made, no further statistical tests were conducted.

### Test phase

A repeated-measures ANOVA was performed with two factors, Prior Predictability (Predictable and Unpredictable) and Correctness (Nil, Correct, or Incorrect). A typical OP effect would manifest as an interaction between these factors (a greater difference between the correct and other outcome ratings for the predictable outcome category than for the unpredictable outcome category). We found a significant main effect of Correctness, *F*(2, 38) = 43.995, *p* < .001, η_p_^2^ = .698, but no significant interaction, *F*(2, 38) < 1, *p* = .754, η_p_^2^ = .013.

### Manipulation check

**Fig. 3:**
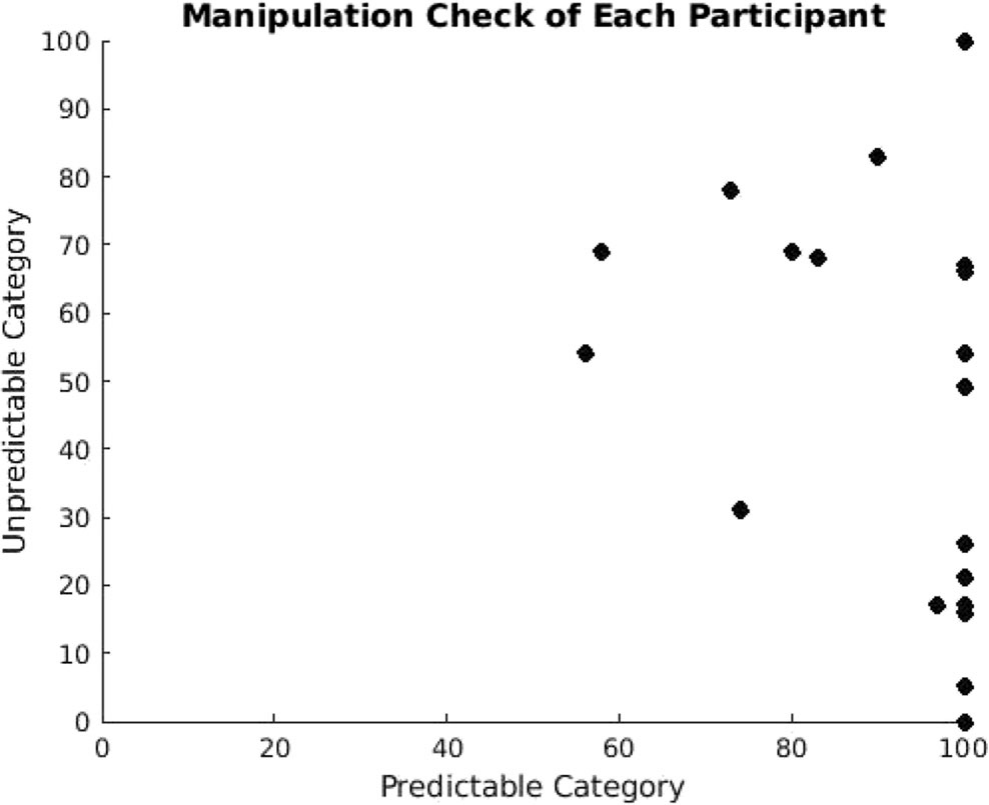
Participants’ ratings during the manipulation check. At the end of the experiment, participants were asked to give a rating between 0 and 100 on how confident they were to predict the allergic reactions of Mr. X in Phase 1. The *x*-axis represents their confidence to predict the predictable category, and the *y*-axis represents their confidence to predict the unpredictable category (0 being the lowest and 100 being the highest level of confidence). The black dots represent each participant’s data point

Participants correctly perceived the predictability of the two outcome categories during the first phase. A paired-samples *t* test resulted in a significant difference between the ratings, as participants gave a higher rating on the predictable category than the unpredictable category (*M*_*predictable*_ *=* 90.55*, M*_*unpredictable*_ *=* 45.55), *t*(19) = 5.432, *p* < .001. Individual data points of the manipulation check are shown in Fig. 3.

## Discussion

We investigated whether learning about unpredictability decreases outcome-specific motivation to learn about an outcome. During Phase 1, people learned to better predict the symptoms from the predictable outcome category than the unpredictable outcome category and later reported the difference in predictability correctly. In Phase 2, participants were asked about which outcome category they would like to learn. Participants were more eager to learn about the predictable outcome first, before learning about the other category in later trials. The motivation to engage with a specific outcome category was therefore affected by its prior predictability. Note that this difference was not a product of that outcome’s current predictability, as all outcomes were equally and perfectly predictable during Phase 2.

This change of outcome-specific motivation to learn could be explained as a result of a change in participants’ expectancy. By the end of Phase 1, participants had the expectancy that they can achieve a successful prediction about the predictable category, but not the unpredictable category. EVT models predict that the expectancy influences motivation to engage. The unpredictability of an outcome during Phase 1 thereby affects the participants’ motivation to learn about that outcome, and therefore their choices in Phase 2. This demotivation to learn could also be a product of frustration felt by being unable to predict correctly, as similar motivational factors have been considered important in animal learning (Amsel, 1962, 1992; Stout et al., 2003). Furthermore, it confirms the similarities with the demotivation caused by the LH effect (Maier & Seligman, 2016). Therefore, this effect could be relevant to a range of mental health disorders such as major depressive disorder (Abramson et al., 1989; Alloy et al., 1992).

Another way to understand these results is through the lens of decision-making processes, particularly risk-taking behavior, and opportunity costs. Risk-taking behavior refers to the preference to take the chances to gain from a choice or an action with an unpredictable outcome (Kacelnik & Bateson, 1996; Platt & Huettel, 2008). In our experiment, a risk-taking behavior would involve choosing to learn about the unpredictable outcome first in Phase 2. However, participants first chose what they had previously known as predictable, even though this behavior creates opportunity costs (i.e., losing a chance to get more information from the other alternative; Anselme, 2015; Kurzban et al., 2013). Participants chose what they knew was predictable and sacrificed the opportunity to learn about the unpredictable category. Once they learned about the previously predictable outcomes and therefore started to make more correct predictions, they increased their risk-taking behavior by choosing the previously unpredictable category. This shifting behavior could be interpreted as a tendency to learn about the other given “opportunity” after learning enough about the firstly chosen one. It indicates that participants changed their evaluation of the opportunity cost they would have to pay when choosing the predictable outcome category again.

This shifting behavior also supports the notion that participants’ choices were more about learning and not about making a correct prediction in each trial. As participants could not make more than one prediction in each trial, they only sacrificed the opportunity to learn about the unpredictable outcomes, but not the opportunity of making more correct predictions when choosing the previously predictable category. Indeed, the best strategy for making the most correct predictions would have been to choose the same category for the entire second phase. Participants nevertheless shifted their choice towards the other category supports the notion that their choice reflects their motivation to learn about an outcome category. This behavior could be related to curiosity. Curiosity is conceptualized as a trait or state of a person, leading to a general desire to learn about the world (see, e.g., Marvin et al., 2020). This desire has two sides: (1) a desire to learn to “know now” and (2) the patience to “wait for later.” One might speculate that the OP, a property of a part of the world, fits the desire to “know now,” and therefore our participants were more eager to learn about the predictable outcome first because they assume that doing so will allow them to acquire information about the new cue–outcome relationships faster.

The results support the proposed link between unpredictability and outcome-specific motivation and offer an account for the OP effect. When participants are confronted with previously unpredictable outcome categories, the reduced outcome-specific motivation might lead to a decreased allocation of cognitive resources for learning about this outcome in new situations, particularly when simultaneously learning about other previously predictable outcomes. In Griffiths et al. (2015) and Thorwart et al. (2017) for example, if participants had the same preference during Phase 2, then despite receiving feedback about both outcome categories, they might devote more resources to using the feedback from the previously predictable outcome. But also when there is only one outcome per trial as in Griffiths et al. (2018), the information about the unpredictable outcomes might be disregarded while prioritized information about predictable outcomes is still processed.

Then why was there no OP effect visible in the test phase of the current experiment? This cannot be due to participants’ opportunity to learn about each cue’s relationship with each outcome category, as the accumulated number of trials at the end of Phase 2 was the same for each cue–outcome combination. However, and in contrast to previous OP experiments using the allergy task (Griffiths et al., 2015; Thorwart et al., 2017), participants were only presented one outcome category in each trial, and it seemed overall easier for the participants to learn about the cue–outcome relationship in the current experiment. Indeed, participants reached about 90% accuracy at the end of Phase 2, which is at least descriptively higher than in previously reported experiments (~80%). Furthermore, as participants had to make an overt choice, this might increase their overall motivation to learn about the chosen outcome category. Therefore, any possible difference in learning about the two outcome categories might not be visible anymore in the test phase.

Some questions exist about whether associative learning is consciously controlled or unconscious and relatively automatic (Frensch & Rünger, 2003; Newell & Shanks, 2014). Since this task is explicit and transparent regarding the goal of making correct predictions, we cannot say with certainty whether participants’ preference for predictable outcomes is driven by a strategic consideration of their goals or a more habitual tendency to choose the more appealing option (the one that has met with the reward of being correct more often). Understanding the locus of these effects is an important consideration for future research.

## Footnotes

1 For simplicity’s sake, we will refer to “unpredictable outcomes” or “unpredictability” even when the outcomes are not completely unpredictable, but only less predictable compared with the fully predictable outcomes.

2 One might think of these allergic reactions as more aversive than rewarding. However, since the aversiveness of the allergic reactions suffered by the fictional patient is independent of the choices that the participant made (correct or incorrect), we did not consider the aversiveness of the allergic outcomes as relevant in this experiment (unlike the relevance of losses vs. gains in reinforcement learning, for instance). In another study, we have investigated the effect of reward (i.e., points won for a correct choice) in the allergy task, which showed that points rewarded in this type of task can also bias learning in future situations (Thorwart et al., 2021).
